# A Comprehensive Analysis of Security Challenges in ZigBee 3.0 Networks

**DOI:** 10.3390/s25154606

**Published:** 2025-07-25

**Authors:** Akbar Ghobakhlou, Duaa Zuhair Al-Hamid, Sara Zandi, James Cato

**Affiliations:** 1 Department of Data Science and Artificial Intelligence, Auckland University of Technology (AUT), Auckland 1010, New Zealand; cato2012@live.com; 2 Department of Computer and Information Sciences, Auckland University of Technology (AUT), Auckland 1010, New Zealand; duaa.alhamid@aut.ac.nz; 3 School of Tech, New Zealand Skills and Education College, Auckland 1010, New Zealand; saraz@nzse.ac.nz

**Keywords:** ZigBee 3.0, denial of service (DoS), Internet of Things (IoT), Centralised Security Model (CSM)

## Abstract

ZigBee, a wireless technology standard for the Internet of Things (IoT) devices based on IEEE 802.15.4, faces significant security challenges that threaten the confidentiality, integrity, and availability of its networks. Despite using 128-bit Advanced Encryption Standard (AES) with symmetric keys for node authentication and data confidentiality, ZigBee’s design constraints, such as low cost and low power, have allowed security issues to persist. While ZigBee 3.0 introduces enhanced security features such as install codes and trust centre link key updates, there remains a lack of empirical research evaluating their effectiveness in real-world deployments. This research addresses the gap by conducting a comprehensive, hardware-based analysis of ZigBee 3.0 networks using XBee 3 radio modules and ZigBee-compatible devices. We investigate the following three core security issues: (a) the security of symmetric keys, focusing on vulnerabilities that could allow attackers to obtain these keys; (b) the impact of compromised symmetric keys on network confidentiality; and (c) susceptibility to Denial-of-Service (DoS) attacks due to insufficient protection mechanisms. Our experiments simulate realistic attack scenarios under both Centralised and Distributed Security Models to assess the protocol’s resilience. The findings reveal that while ZigBee 3.0 improves upon earlier versions, certain vulnerabilities remain exploitable. We also propose practical security controls and best practices to mitigate these attacks and enhance network security. This work contributes novel insights into the operational security of ZigBee 3.0, offering guidance for secure IoT deployments and advancing the understanding of protocol-level defences in constrained environments.

## 1. Introduction

The IoT represents a rapidly expanding technological domain, with ZigBee playing a pivotal role in enabling wireless personal area networks (WPANs) for diverse IoT applications. Built upon the IEEE 802.15.4 specification, ZigBee facilitates connectivity among a wide array of devices through mesh, star, and tree topologies. Its design prioritises low data rates, minimal power consumption, and cost-efficiency, making it suitable for a broad spectrum of devices, including battery-operated wireless sensor nodes with extended battery life [1]. ZigBee has secured a significant market share, particularly in smart home, industrial, and healthcare applications [2].

The increasing demand for smart home applications has positioned ZigBee as a leading standard in home automation. Major manufacturers such as Amazon, Samsung, and Philips have integrated ZigBee into household appliances, enabling remote monitoring and control via the internet [3]. Beyond smart homes, ZigBee is employed in various domains, including industrial automation and healthcare, where it supports wireless sensor nodes (WSNs) in monitoring and collecting environmental data [4].

Given its deployment in data-sensitive applications, ZigBee incorporates several security controls to ensure data confidentiality, integrity, and authentication. The protocol employs a simplified version of the 128-bit AES for encryption, leveraging the built-in security features of the IEEE 802.15.4 standard. These measures are crucial for protecting sensitive data, such as patient information in healthcare and personal data in smart homes [5]. Despite these security measures, ZigBee’s low-cost, low-power design introduces vulnerabilities that have persisted across protocol revisions, raising concerns about its overall security.

The main contributions of this work are summarised as follows:

- Investigation of security issues in ZigBee 3.0: This study addresses the gap in the literature by examining the prevalent security issues in ZigBee 3.0 networks.
- Focus on core security concerns: The research specifically investigates the following three core security concerns: (a) the security of symmetric keys, (b) the implications of compromised symmetric keys, and (c) the adequacy of DoS protection mechanisms.
- Impact on network security: The study evaluates the impact of identified security issues on the overall security of ZigBee 3.0 networks.
- Enhancement of protocol robustness: This study demonstrates how ZigBee 3.0’s security features, such as install code-based authentication, trust centre link key updates, and Secure Remote Password (SRP) authentication, can be effectively configured to mitigate key compromise and denial-of-service attacks. Through real-world testing we show that these mechanisms significantly improve the protocol’s resilience and operational robustness in practical IoT deployments.
- Contribution to IoT security knowledge: The findings of this study add to the broader understanding of IoT security, particularly in the context of widely used standards like ZigBee.

The remainder of this paper is structured as follows: Section 2 discusses state-of-the-art and related work. Section 3 presents the system model. Section 4 evaluates the testbed based on the proposed use cases. Finally, in Section 5, the conclusion of the work is discussed.

## 2. Related Work

As information technology advances, the rapid expansion of IoT devices is evident. ZigBee, a short-range wireless communication standard utilised in IoT devices, is expected to find applications in smart homes and industrial control systems due to its efficiency in terms of low power consumption and cost-effective operation, despite its comparatively slower communication speed. Nonetheless, ZigBee is vulnerable to cyber attacks, as eavesdropping on packets and the transmission of forged packets in wireless communication pose greater challenges compared to wired alternatives. Many studies attempt to outline various techniques for detecting the types of attacks that may arise in ZigBee-based IoT systems [5,6,7].

In response to these vulnerabilities, the security of ZigBee networks relies on symmetric cryptography with a pre-shared secret. In the current Zigbee protocol, when establishing a network the network coordinator generates a network key. This key is securely distributed to joining devices, encrypted with the pre-shared secret, to protect future communications. However, the necessity of pre-installing the secret in millions of devices before deployment poses a risk of potential leaks, compromising the network’s confidentiality and integrity [8]. The authors of Ref. [8] introduced an advanced certificate-less joining protocol to enhance Zigbee network security. This protocol employs cost-effective public key primitives and consists of two main components. First, it integrates the Elliptic Curve Diffie–Hellman key exchange into existing association request/response messages, facilitating both link-to-link communication and encryption of the network key to enhance user device privacy. Second, it improves the security of the installation code, a novel ZigBee 3.0 joining method, using public key encryption. ProVerif formal verification methods confirmed the protocol’s robustness, and a prototype developed with open-source software and hardware demonstrated its efficiency and effectiveness. According to the authors, the new protocol introduced no additional messages, with an average overhead as low as 3.8% during the join procedure. In a recent study, the authors of Ref. [9] developed Verejoin, an automated verification tool, to systematically analyse the rejoin procedure in Zigbee networks. The tool not only validated a well-known design flaw but also uncovered two previously unknown design flaws. Furthermore, the researchers created four proof-of-concept attacks to exploit these vulnerabilities, revealing new attack surfaces for the potential manipulation of Zigbee devices. The impact of these vulnerabilities ranges from denial of service to device hijacking. To further validate their findings, the researchers designed ZigHomer, a Zigbee testing tool. Using ZigHomer, they extensively evaluated off-the-shelf Zigbee devices from major IoT vendors, uncovering the widespread prevalence and severity of these vulnerabilities. The researchers reported their findings to relevant parties, all of whom acknowledged the substantial security impact [9]. Additionally, the authors of Ref. [10] conducted a broader security analysis of ZigBee, identifying further attack vectors and reinforcing the need for robust security mechanisms in ZigBee-based IoT systems.

The ZigBee protocol employs the AES for data security, but concerns exist about its vulnerability to future attacks. Additionally, symmetric cryptosystems, like AES, face challenges in key management and authentication [11]. To address these issues in ZigBee communications, the authors of Ref. [12] proposed a mutual authentication scheme that dynamically updates secret key values for device-to-trust centre and device-to-device communications. The solution enhanced cryptographic strength without relying on asymmetric cryptography, utilising secure one-way hash functions and bitwise exclusive OR operations during mutual authentication. Once authenticated, ZigBee participants agree on a shared session key, exchanging a secure value integrated with sensed data for regular AES encryption [12].

ZigBee networks, widely used in IoT applications, face significant security challenges, particularly in key management. Protocol weaknesses provide opportunities for attackers to exploit vulnerabilities, leading to potentially harmful attacks. A major concern is the extraction of encryption keys, which can facilitate malicious activities [13,14,15,16,17,18]. A passive eavesdropper can intercept and analyse transmitted data, potentially extracting the network key without detection. These risks are made worse by the lack of dynamic key negotiation, key rotation, or strong mutual authentication. As a result, Zigbee’s key management architecture reduces the overall security of deployments, especially in large-scale or high-risk IoT environments.

In summary, while ZigBee’s security architecture incorporates several layers of protection through its MAC, NWK, and APL layers, it is not without its vulnerabilities. The protocol’s reliance on symmetric key cryptography and its low-cost, low-power design have historically left it susceptible to various security threats, including key compromise and denial-of-service attacks. Recognising these challenges, the ZigBee Alliance introduced ZigBee 3.0, a significant update aimed at enhancing the protocol’s security features.

The literature review on ZigBee 3.0 revealed several key points and notable research gaps. ZigBee is a widely used wireless technology standard for IoT devices, known for its low power consumption and cost-effectiveness. It is implemented in various applications, including smart homes, industrial automation, and healthcare [19]. However, it continues to face security issues, particularly in symmetric key management, such as the use of default link keys and unencrypted network key transport. Additionally, ZigBee remains vulnerable to various DoS attacks due to inherent protocol limitations and a lack of robust protective measures [20]. ZigBee 3.0 addresses some of these concerns by introducing mandatory trust centre link key updates and the optional use of install codes for device authentication, thereby enhancing overall security [21].

Despite extensive research on ZigBee’s vulnerabilities, most studies rely on simulations or theoretical models. There is limited empirical evaluation of ZigBee 3.0’s updated security features, such as install codes, trust centre link key updates, and SRP authentication, in real-world deployments. Moreover, the comparative impact of Centralised vs. Distributed Security Models on protocol resilience remains underexplored. This study addresses these gaps through practical experimentation and analysis.

## 3. System Model

The system model for assessing the security of ZigBee 3.0 networks is designed to evaluate the impact of prevalent security issues related to symmetric keys and DoS attacks. The model is structured around a series of practical security testing experiments conducted in a controlled environment. The security testing framework begins with planning, which establishes the groundwork for security tests, including defining the steps of engagement and configuring the ZigBee network. The discovery phase involves gathering information from the network to identify potential vulnerabilities. This is followed by the attack phase, where the planned attacks are executed to test the network’s security. Finally, the reporting phase documents the findings and impact of the attacks.

**Figure 1:**
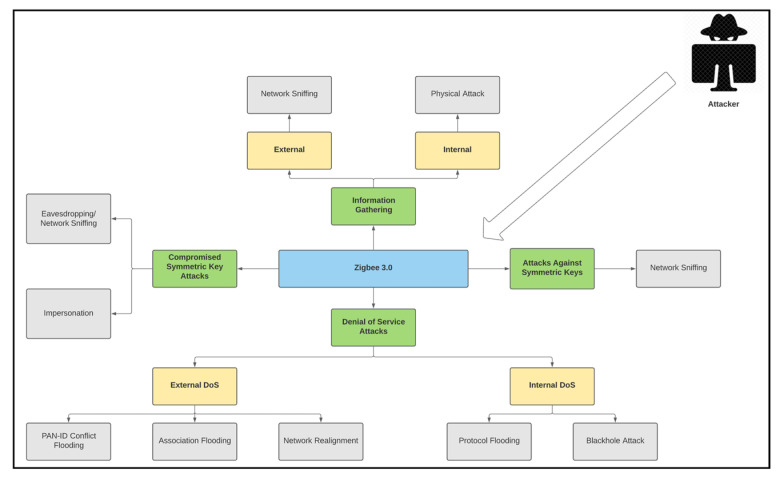
ZigBee symmetric key and DoS attack model.

The system model addresses security issues and attack models, focusing on the security of symmetric keys and the network’s resilience against DoS attacks. It evaluates vulnerabilities such as unencrypted network key transport and default link key values, and assesses the impact of exposed symmetric keys on network confidentiality. The model also tests the network’s resilience against various DoS attacks, both internal and external, as explained in Figure 1.

**Figure 2:**
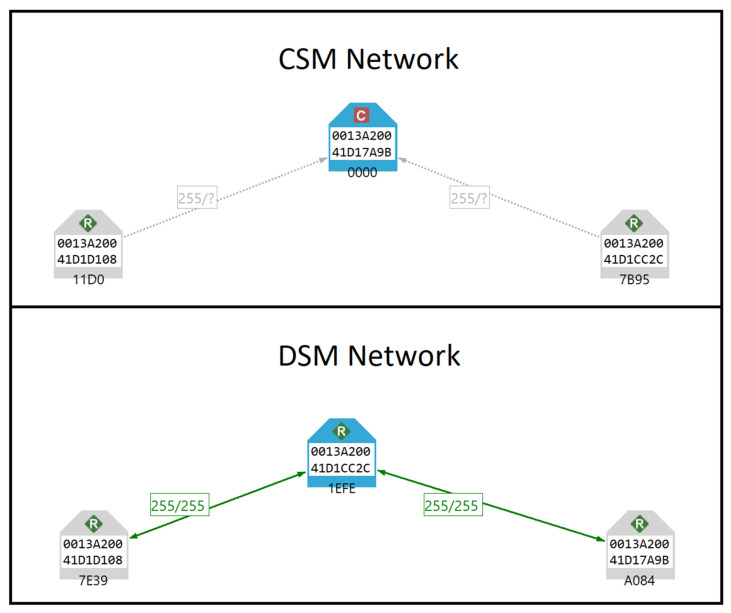
ZigBee 3.0 security models shown in XCTU network scan.

The testing environments include both the Centralised Security Model (CSM) and the Distributed Security Model (DSM). In the CSM, a single trust centre manages the network’s security, while in the DSM, multiple routers can act as trust centres, simplifying device authentication but with certain security trade-offs, as explained in Figure 2.

These two configurations were selected because they represent the two security models outlined in the Zigbee 3.0 specification and reflect real-world deployment scenarios. CSM is commonly used in settings where stronger, centralised trust management is needed, such as healthcare. On the other hand, DSM is more suitable for flexible or low-power deployments where centralised management is not feasible. By including both models, we were able to evaluate how security mechanisms, particularly those related to key distribution and trust, perform under differing operational conditions and explore what Zigbee 3.0 offers. Other aspects of the network configuration were held constant to ensure controlled and meaningful comparison between the two models. Additionally, to ensure consistency across experiments, a common ‘base configuration’ was applied to the testbed networks. This included enabling key security features recommended by Zigbee 3.0, such as encryption and device authentication. However, default values were retained for certain configurable parameters when they reflected realistic out-of-the-box deployments or allowed us to focus on the impact of the specific security model being tested. This approach ensured that the comparison between CSM and DSM configurations remained focused on differences in trust and key management, without introducing variability from unrelated protocol settings.

Additionally, all experiments were conducted in a controlled indoor laboratory environment with minimal RF interference. No other ZigBee or IoT networks were active in proximity during testing. Zigbee 3.0 testbed networks were formed using automatic channel selection, allowing the protocol to choose the least congested channel available. Although interference levels were not quantitatively measured, care was taken to ensure that no active interference sources were present and that the environment remained consistent across all experiments to minimise variability in results.

The ZigBee 3.0 network configuration involves both hardware and software setups that will be discussed in the next sub-section. The hardware setup utilises XBee 3 Pro modules, development boards, and Waspmote v1.5 development boards to construct the network. The software setup employs XCTU software for configuration and monitoring, and Waspmote IDE for programming end devices. The base configuration defines the initial settings for network formation, device joining, and security policies.

Data collection and analysis are conducted using both qualitative and quantitative methods. Qualitative methods involve observing and interpreting the impact of attacks through direct observation and network scans. Quantitative methods measure the effect of attacks numerically, such as the number of packets received or dropped during DoS attacks. This comprehensive assessment ensures a robust evaluation of ZigBee 3.0’s security posture by systematically testing and analysing its vulnerabilities and resilience to attacks.

### 3.1. ZigBee Hardware and Software Setup

#### 3.1.1. ZigBee Hardware Setup

The selected hardware for this research was well-suited due to its flexibility in configuring and programming devices to meet the specific requirements of each experiment. Table 1 provides an overview of the hardware components used to construct each individual node.

**Figure 3:**
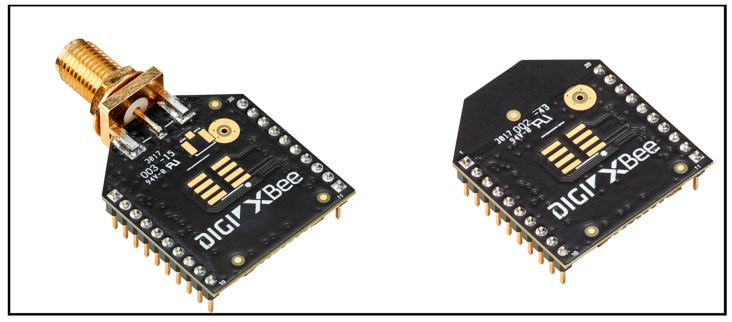
XBee 3 modules.

**Figure 4:**
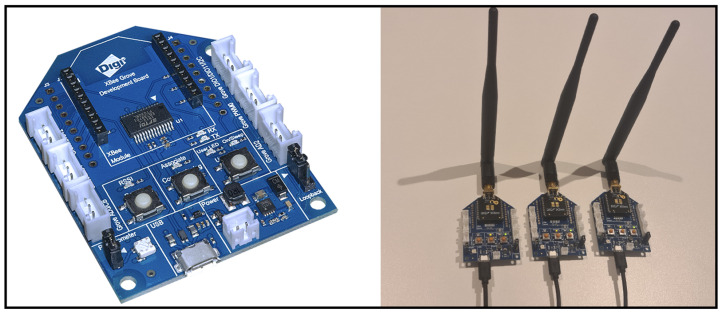
XBee development board and gateway nodes.

**Figure 5:**
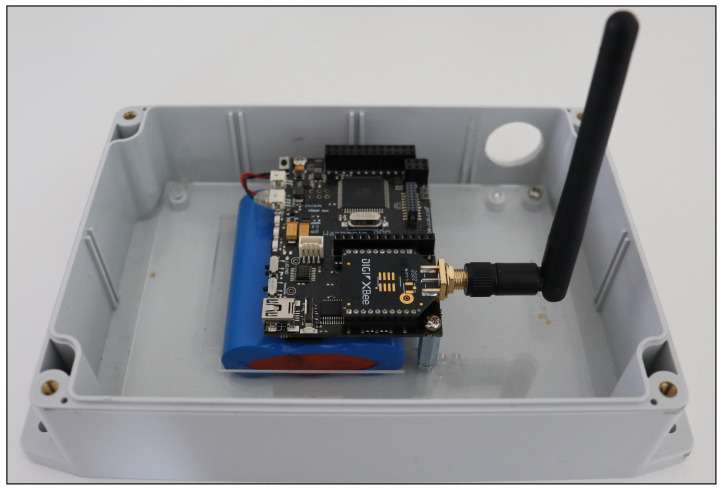
End device (Waspmote) node.

- Computer (Windows 10): A desktop computer running Windows 10 was used to configure, monitor, and maintain the ZigBee 3.0 testbed networks. The coordinator and router nodes were connected to the computer’s USB ports, establishing a gateway between the ZigBee network and the PC.
- XBee 3 modules: A total of five XBee 3 Pro (XB3-24Z8ST) and three XBee 3 (XB3-24Z8PT-J) modules were used. These radio modules allowed the supported hardware to operate as coordinator, router, or end device nodes through the configured Application Programming Interface (API) mode. The XBee 3 modules are integrated with the ZigBee 3.0 protocol and operate on the ISM 2.4 GHz frequency, ensuring compatibility with several third-party devices. XBee 3 modules are shown in Figure 3.

- XBee development boards: Three XBee Grove Development Boards were employed to construct the gateway nodes. These boards facilitated data flow between the ZigBee network and the PC via a standard USB port, acting as a data bridge or access point. The XBee 3 modules were connected to the boards through grove connectors, enabling evaluation through the XCTU software. XBee development board and gateway nodes are shown in Figure 4.

- Waspmote v1.5 development boards: The Waspmote v1.5 boards, designed for integration with various IoT technologies including ZigBee, were used to create up to five end device nodes. These boards operated on battery packs and were programmed via the Waspmote IDE software, allowing them to function autonomously. The end device (Waspmote) node is shown in Figure 5.

This hardware setup provided a robust and adaptable foundation for conducting the security testing experiments, ensuring that the ZigBee 3.0 networks could be effectively configured and monitored throughout the research.

#### 3.1.2. ZigBee Software Setup

**Figure 6:**
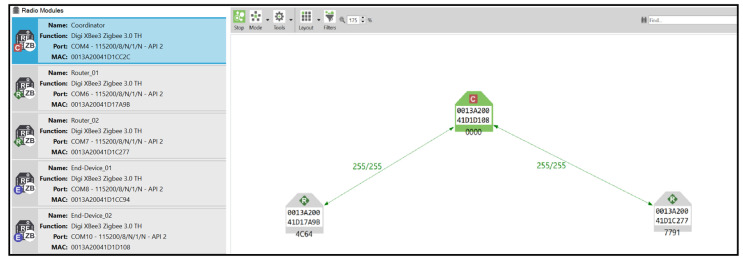
XCTU locally connected modules and network scan.

The software implementation for this research is critical for configuring, maintaining, and monitoring the ZigBee 3.0 networks. The following software tools were utilised:

- XCTU: Digi XCTU is a comprehensive configuration and test utility software essential for updating, configuring, and managing the XBee 3 radio modules. XCTU facilitates the setting of node parameters, including network security configurations and policies, and other essential settings that determine node communication. The software connects locally to the XBee 3 modules via the USB interface of the XBee development boards. XCTU’s network scan function is particularly valuable for internal network monitoring, providing a graphical display of nodes, pathways, and their respective signal strengths. This function was employed to verify network formation and monitor changes during experiments. Additionally, XCTU’s frames generator tool was used to create and send custom API frames from locally connected XBee 3 modules, enabling the creation of any frame supported by the ZigBee 3.0 protocol and facilitating internal attack verification. Figure 6 shows an XCTU locally connected modules and network scan.

- Waspmote IDE: The Waspmote IDE software was used to programme the Waspmote v1.5 development boards, enabling the end device nodes to operate autonomously. The IDE includes a library of example codes that were modified and uploaded to the Waspmote boards, ensuring seamless integration and functionality within the ZigBee 3.0 networks.

These software tools were integral to the successful implementation and analysis of the ZigBee 3.0 networks, providing robust capabilities for configuration, monitoring, and experimentation.

### 3.2. Security Testing Setup

This section details the hardware and software employed to conduct security testing experiments on ZigBee 3.0 networks (see Table 2).

#### 3.2.1. Security Testing Hardware

**Figure 7:**
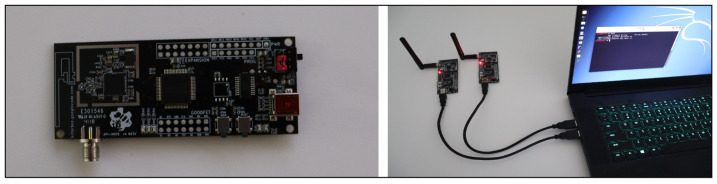
ApiMote v4 and research laptop.

**Figure 8:**
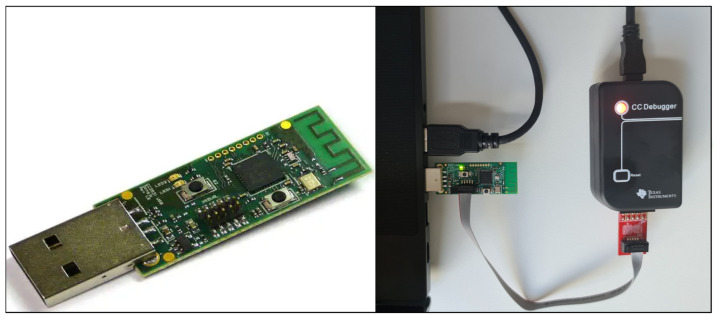
CC2531 USB dongle and Flashing ZBOSS Firmware with CC Debugger.

- Research laptop: A standard laptop running Kali Linux OS was used to perform external attacks. It hosted the necessary software for conducting security tests and monitoring the ZigBee networks.
- ApiMote: This ZigBee security research hardware, designed by River Loop Security, was used for sniffing and injecting packets on ZigBee/IEEE 802.15.4 networks. Two ApiMote v.4 BETAs were used to capture and inject packets simultaneously. Each ApiMote was equipped with a screw-on antenna for optimal range and connected to the research laptop via mini-USB. Figure 7 shows the ApiMote v4 and research laptop.

- CC2531 USB dongle: This device, flashed with ZBOSS sniffer firmware, was used to capture ZigBee network traffic over Wireshark. The CC2531 USB dongle and flashing ZBOSS firmware with CC Debugger are shown in Figure 8.

- Compromised nodes: In certain experiments, attacks were initiated from within the network using compromised nodes. These nodes, consisting of an XBee 3 module and an XBee development board, contained the network and security configuration of the victim network, allowing them to perform internal attacks.

#### 3.2.2. Security Testing Software

- Kali Linux 2018-3: The external attack experiments were conducted using a Kali Linux virtual machine (version 2018.3), chosen for its preconfigured software dependencies required to run the KillerBee framework and Wireshark.
- KillerBee framework: This open-source, python-based framework was used for exploring and evaluating the security of ZigBee and IEEE 802.15.4 networks. It included various tools for sniffing and injecting packets.
- Wireshark: This packet analysing tool was extensively used for network and packet analysis. It captured packets across ZigBee/IEEE 802.15.4 specified channels through a ZBOSS flashed CC2541 USB interface. Capture sessions were saved as ‘.pcap’ files for further analysis.
- XCTU: This configuration and test utility software was used to update, configure, and manage the XBee 3 radio modules. It facilitated setting node parameters, including network security configurations and policies, and provided a graphical display of nodes and their respective signal strengths.

These components were integral to the successful implementation and analysis of the ZigBee 3.0 networks, providing robust capabilities for configuration, monitoring, and experimentation.

## 4. Model Testing and Evaluation

This section details the testing and evaluation methodologies employed to assess the security robustness of ZigBee 3.0 against known vulnerabilities from previous versions. The findings from the security testing experiments are presented, highlighting the impact of prevalent security issues on ZigBee 3.0. Preliminary experiments focus on information gathering techniques used during the discovery phase to collect essential data for subsequent exploitation of ZigBee 3.0. The security of symmetric keys is discussed, examining known vulnerabilities and proposing methods to enhance key security in ZigBee 3.0. The consequences of compromised symmetric keys on ZigBee 3.0 networks are evaluated. Finally, the results of various DoS attacks are presented, assessing ZigBee’s resilience against such threats.

### 4.1. Information Gathering on ZigBee 3.0

This outlines the methodologies employed for information gathering on ZigBee 3.0 networks, which are crucial for subsequent exploitation. The experiments conducted are divided into two categories: external and internal/physical information gathering.

#### 4.1.1. External Information Gathering

**Figure 9:**
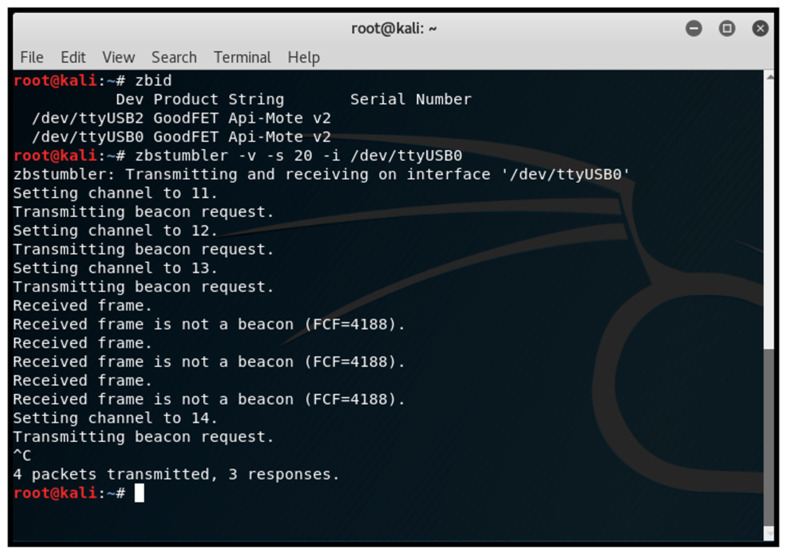
Discovering ZigBee network’s operating channel with KillerBee.

**Figure 10:**
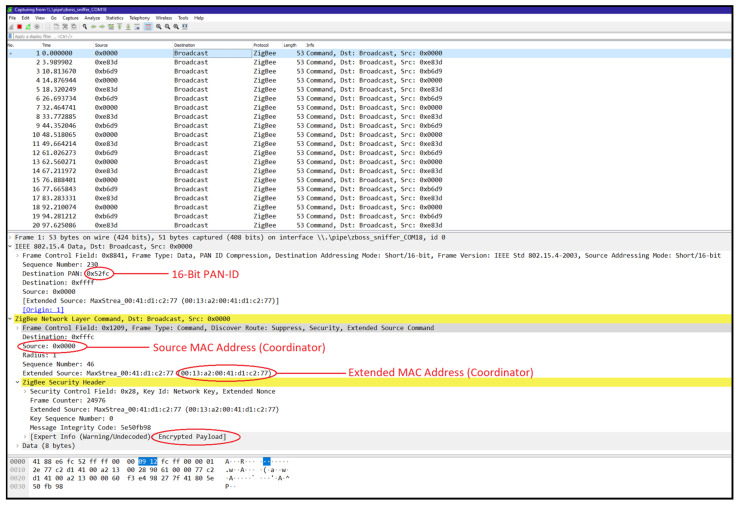
Obtaining network information over Wireshark.

**Figure 11:**
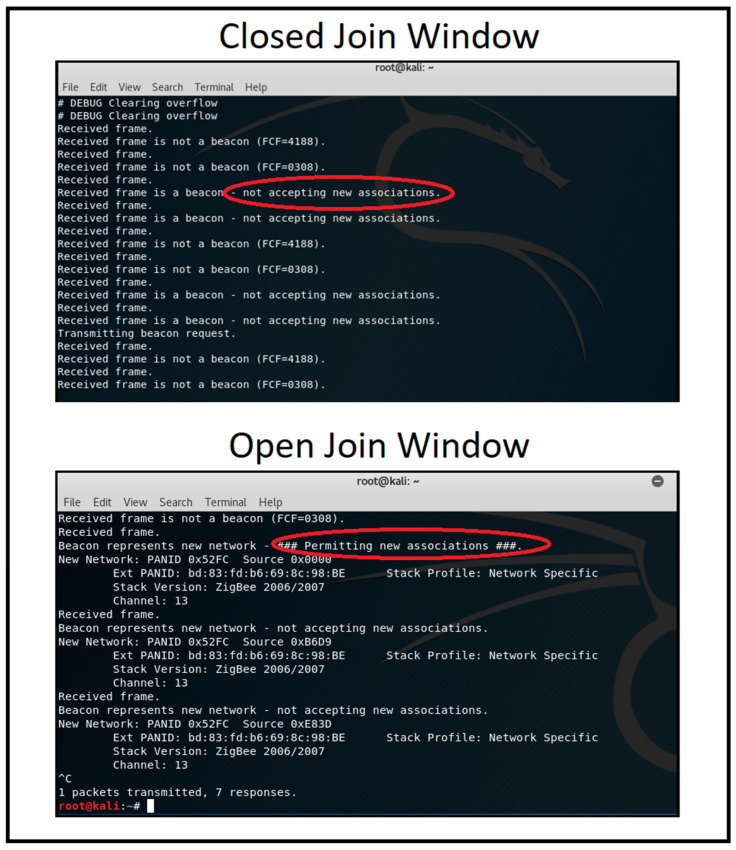
Monitoring ZigBee join window.

External information gathering involves interacting with the ZigBee network from outside to collect vital network information. The following steps were undertaken:

1. Network discovery and operating channel identification: We utilised the zbstumbler tool to send beacon requests across channels, identifying the operating channel when a ZigBee device responded. As shown in Figure 9, the tool zbstumbler was executed, and a response was received on channel 13, indicating an active ZigBee network.
2. Network sniffing: We employed a CC2531 USB dongle with ZBOSS firmware to capture network traffic using Wireshark. This process revealed the source and extended MAC addresses, 16-bit PAN-ID, and indicated that the payload was encrypted with a network key. Figure 10 shows the ZigBee broadcast communications captured, revealing the source and extended MAC addresses and 16-bit PAN-ID. The MAC addresses of end devices were discovered in various data packets sent from the device.
3. Monitoring join window: We monitored the network’s join window state using the zbstumbler tool, which indicated when the join window was open or closed on gateway nodes. Figure 11 shows the join window state being monitored, indicating that the join window is closed on the gateway nodes.

#### 4.1.2. Internal/Physical Information Gathering

**Figure 12:**
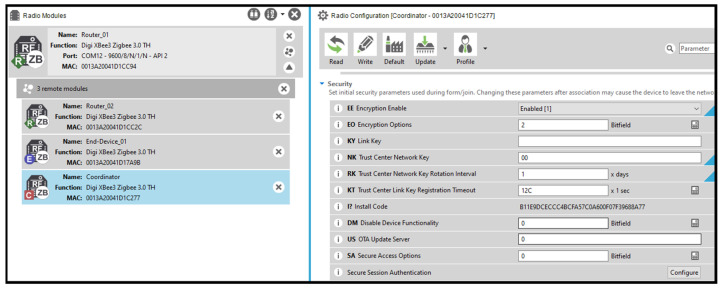
Remotely accessing coordinators’ configuration.

Internal information gathering assumes physical access to a ZigBee device, allowing direct interaction with its configuration parameters:

1. Compromised end device node: We connected the compromised end device to XCTU software, revealing all configuration parameters except for the symmetric keys, which are write-only.
2. Compromised router node and remote coordinator access: Similarly to the end device, the router node’s configuration parameters were accessible. Additionally, the router could remotely access and configure the coordinator node’s settings, including security configurations. Figure 12 shows the compromised router remotely connecting to the coordinator node and accessing its security configuration.

These methodologies provide a comprehensive foundation for further exploitation of ZigBee 3.0 networks, ensuring a thorough understanding of the network’s structure and security configurations.

### 4.2. Securing Symmetric Keys with Install Codes

Within ZigBee 3.0, devices offer a secure network entry option through the utilisation of install codes. This mechanism allows devices to join the network in a manner that ensures heightened security for symmetric keys. Notably, the utilisation of install codes guarantees the provision of distinct random link keys for each joining device, thereby obviating the need for authentication via a global link key.

As demonstrated in our research, the procedure entails the individual registration of a joining node to the trust centre. This registration is orchestrated with the aid of a random link key derived from the device’s unique install code. This strategic approach stands out as a beacon of security for symmetric keys. It enforces the creation of entirely randomised link keys for every device, thus creating a robust safeguard against potential vulnerabilities stemming from compromised global or default link keys.

**Figure 13:**
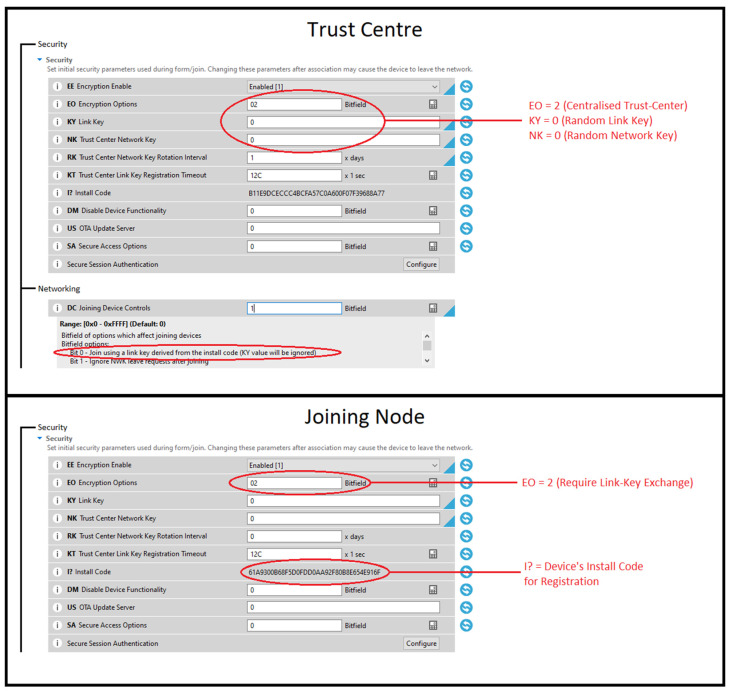
ZigBee 3.0 install code joining configuration on XBee 3.

In essence, this technique fortifies the security of ZigBee 3.0 networks by ensuring that each device’s link key remains devoid of predictability or pattern. By eliminating the reliance on shared global keys, the network key is shielded from potential exposure. This pivotal practice contributes to the establishment of a resilient and secure network architecture, amplifying the overall protection against unauthorised access and potential key-based exploits. Figure 13 illustrates the configuration applied to the joining node and trust centre to enable joining via install codes.

### 4.3. Node Impersonation Attacks

In our experiment, we explored a node impersonation attack targeting ZigBee 3.0 networks, leveraging compromised symmetric keys. In this scenario, the attacker assumed the role of a legitimate coordinator node, utilising the acquired network information and compromised symmetric keys of the victim network. To conduct the node impersonation attack, we configured an impersonated coordinator by employing the compromised symmetric keys (with the exception of the updated trust centre link key). The objective was to hijack a victim node from the targeted network. During the experiment, the attacker sent a spoofed coordinator realignment frame to a victim node while the join window of the impersonating coordinator was open. The attack outcomes varied based on the network configuration.

1. Against the CSM network: The victim node left its initial network but did not join the attacker’s node.
2. On the DSM network: The victim node left its initial network and successfully joined the impersonated coordinator network.

The success of the node impersonation attack on the DSM network indicates the potential risk of unauthorised node inclusion, which may lead to data being compromised or even a DoS situation.

**Figure 14:**
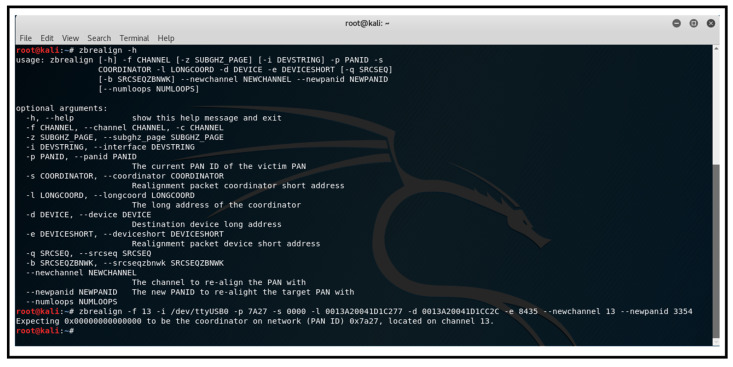
Executing ‘zbrealign’ script from Kali Linux.

Figure 14 shows the execution of the zbrealign tool to create and send a spoofed coordinator realignment frame to the victim network.

It is essential to emphasise that this experiment was performed under the assumption that the attacker had already acquired the necessary network information and compromised the symmetric keys of the targeted network.

### 4.4. Denial-of-Service Attacks

Our DoS experiments aimed to assess the effectiveness of DoS protection mechanisms within ZigBee 3.0. This was accomplished by conducting a sequence of attacks and gauging their influence on network and service availability. The experiments involved both internal and external DoS attacks on a singular CSM network. To evaluate the impact, we utilised both qualitative observations and quantitative measurements.

- Security configuration and setup: The DoS attack experiments were conducted against a single ZigBee 3.0 CSM network consisting of eight nodes, including three gateway nodes and five end device nodes. The network employed a centralised trust centre ((encryption options (EO)) = 2), and each device was initially authenticated with a preconfigured link key.
- End device functionality: Each end device node was programmed to report its battery level by generating and transmitting a packet to a router (gateway) node every 3 s. The programme uploaded to each Waspmote v.1.5 Board was a modified ‘XBee’ communications example code sourced from the Waspmote IDE.
- Gateway functionality: The gateway nodes passively relayed messages between nodes throughout the network. In certain experiments, a router node was configured to transmit packets to the other router node at predefined intervals.

#### 4.4.1. External DoS Attacks

The external DoS attacks were executed externally against the network using the Kali Linux machine and ApiMote hardware to exploit the functionality of the ZigBee protocol, aiming to impact the availability of the testbed network and its services. The PAN-ID flooding attack utilised KillerBee tools and ApiMote hardware to rapidly generate and transmit spoofed packets, leveraging the acquired network information across the victim network’s operating channel. The network realignment attack exploited the protocol by sending a spoofed realignment packet, attempting to isolate a victim node from its network.

##### PAN-ID Conflict Flood

**Figure 15:**
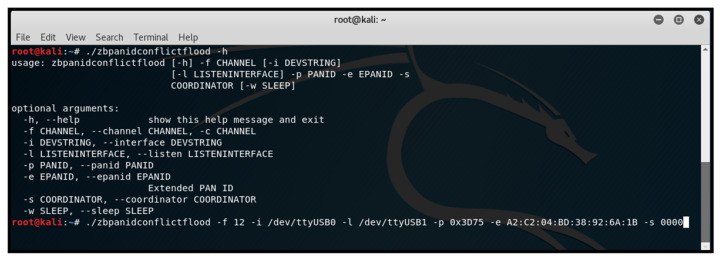
Executing ‘zbpanidconflictflood’ script in Kali Linux.

In ZigBee networks, a node responds to a beacon request frame with the same 16-bit PAN-ID by reporting a PAN-ID conflict to the network manager (Coordinator). In our research, we exploited this functionality on the ZigBee 3.0 network using the ‘zbpanidconflictflood’ tool from KillerBee, in conjunction with two ApiMote interfaces. Figure 15 shows us executing the ‘zbpanidconflictflood’ script in Kali Linux.

PAN-ID conflict flooding was conducted, resulting in a limited-to-moderate impact on the network. This impact was observed as slight delays in the received rate on router nodes. No dropped packets were observed, although some nodes experienced delayed intervals during PAN-ID changes. The ability of router nodes to perform XCTU network scans was affected, requiring alignment with coordinators for initiation. Device authentication at the trust centre remained unaffected. Extended testing spanning 12 h showed no changes to the initial routing structure or disruptions in node/network operations. The effectiveness of the ‘PAN Conflict Threshold’ setting was demonstrated, allowing control over PAN-ID changes for minimal network and node impact. Disabling the PAN-ID change feature entirely was also explored.

##### Network Realignment Attack

The network realignment attack aimed to disrupt the functioning of a specific node within the ZigBee network. The objective was to manipulate the operational parameters of the targeted node, which, in this instance, was referred to as ‘Router_02’. This manipulation caused the node to become detached from its network, resulting in a situation of DoS attacks. The attack achieved this by reconfiguring the target node to adopt a new PAN-ID, thereby disrupting its routing and data reception capabilities. Consequently, this action led to the disconnection of the node’s associated child nodes from the network.

Through the deployment of a spoofed coordinator realignment frame, the network realignment attack produced a noteworthy DoS effect on the designated node. Upon receipt of the falsified frame, the victim router node swiftly disengaged from the network and adjusted its 16-bit PAN-ID to align with the realignment instructions. Furthermore, this disruption led to the departure of two out of the four initially linked child end device nodes from the network. These child nodes could only rejoin the network once the join window opened, and the victim router node was left in an isolated state. Resolution necessitated the resetting of networking parameters and re-registration of the victim node within the network.

#### 4.4.2. Internal DoS Attacks

The examination of internal DoS attacks within the ZigBee 3.0 network were explored. These attacks are executed by leveraging a compromised router node, denoted as ‘Router_02’, which is inherently integrated into the network structure.

The first experiment, titled ‘Protocol Flooding’, constitutes a flooding attack where a solitary node is bombarded with valid messages, each featuring the maximum payload size that the system supports. The subsequent experiment exploits remote AT commands, effectively triggering a DoS condition that directly affects the victim coordinator. These experiments collectively analyse and assess internal attack scenarios, all of which aimed to undermine the network’s availability.

##### Protocol Flooding

Protocol flooding involved bombarding a target router node with legitimate packets of ZigBee’s maximum payload size, originating from a compromised router node. This deliberate barrage induced notable disruptions in processing and routing within the victim node.

An assessment revealed significant delays in packet reception at the victim router, with a majority of packets experiencing delivery failures during the attack’s course. Interestingly, an extended attack spanning 12 h demonstrated that the network’s foundational routing structure remained unaffected, and no instances of node crashes were observed despite the continuous assault.

##### Blackhole Attack Using Remote at Commands

Remote AT commands, a feature within the ZigBee protocol designed for configuring devices from a distance, were exploited in an experiment to induce a DoS scenario in a ZigBee 3.0 network. The aim of this attack was to reset the networking parameters of a target coordinator, causing disruption to routing paths for its associated child nodes and resulting in the rejection of their transmitted packets.

In this context, a ‘network reset’ command was employed as part of a blackhole attack. This involved crafting and transmitting the command from a compromised router node to the coordinator node of the targeted network. Upon reception, the coordinator’s networking parameters, encompassing both 16-bit and 64-bit PAN-IDs, along with the operating channel, underwent a reset. As a result, an entirely new network structure was formed and the initially connected child end device nodes adjusted accordingly.

This disruption severed the routing path for packets sent from connected end device nodes to the router nodes, leading to the discarding of these packets. An important observation was that XBee 3 modules had the capability to counter unauthorised remote AT commands through SRP authentication. By enabling SRP, nodes could establish secure sessions for remotely executing AT commands, ensuring a higher level of command control.

## 5. Conclusions

This study comprehensively evaluated the ZigBee 3.0 protocol against prevalent security issues identified in earlier revisions, focusing on symmetric key vulnerabilities and DoS attacks. The findings revealed that ZigBee 3.0 has significantly improved its security mechanisms, particularly in safeguarding symmetric keys. The protocol’s default configurations effectively mitigate known vulnerabilities such as unencrypted network key transport and default link key values. Additionally, the introduction of install code-based device registration and mandatory trust centre link key updates in the CSM further enhance the security of symmetric keys.

Despite these advancements, the study identified that ZigBee 3.0 remains susceptible to specific DoS attacks, including network realignment, protocol flooding, and remote AT command exploitation. These attacks primarily result in data loss and disruptions to network availability. The research underscores the importance of employing robust security configurations and best practices, such as using install codes for device registration and enabling SRP authentication, to mitigate these risks.

This work advances the current understanding of ZigBee 3.0 security by providing empirical evidence from real-world deployments, highlighting the practical implications of protocol-level vulnerabilities and the effectiveness of newly introduced security features. The comparative analysis of Centralised and Distributed Security Models offers valuable insights into their respective strengths and limitations in operational environments. By bridging the gap between theoretical models and practical experimentation, this study contributes to the developments of more secure and resilient ZigBee-based IoT systems. Future research should explore additional attack vectors and the impact of integrating legacy devices into ZigBee 3.0 networks to further enhance the protocol’s resilience. In addition, we plan to explore the use of Artificial Intelligence (AI) tools to support the detection and analysis of vulnerabilities in ZigBee and similar radio protocols. Machine learning techniques could help identify abnormal traffic patterns, automate attack detection, and improve the efficiency of security testing in wireless networks.

## Figures and Tables

**Table 1:** ZigBee node hardware.

Coordinator Node	Router Node	End Device Node
XBee 3 Pro module	XBee 3 Pro module	XBee 3/XBee 3 Pro modules
XBee development board	XBee development board	Waspmote v1.5 development board
Antenna	Antenna	Antenna

**Table 2:** Security testing hardware and software.

Component	External Attacks/Network Analysis	Internal Attacks (Compromised Node)
Hardware	Laptop (Kali Linux). 2x ApiMote (flashed with KillerBee) with Antenna. 1x CC2531 USB dongle (flashed with ZBOSS).	XBee 3 Pro. XBee development board. Antenna.
Software	Kali Linux 2018-3. KillerBee. Wireshark (ZBOSS).	XCTU.

## Data Availability

The original contributions presented in this study are included in the article. Further inquiries can be directed to the corresponding author(s).
